# The neurodevelopmental implications of hypoplastic left heart syndrome in the fetus

**DOI:** 10.1017/S1047951116001645

**Published:** 2016-11-08

**Authors:** David F. A. Lloyd, Mary A. Rutherford, John M. Simpson, Reza Razavi

**Affiliations:** 1 Paediatric Cardiology Department, Evelina Children’s Hospital; 2 Division of Imaging Sciences and Biomedical Engineering, King’s College London, London, United Kingdom

**Keywords:** Fetal cardiology, paediatric cardiology, CHD, hypoplastic left heart syndrome, neurodevelopment

## Abstract

Abstract As survival after cardiac surgery continues to improve, an increasing number of patients with hypoplastic left heart syndrome are reaching school age and beyond, with growing recognition of the wide range of neurodevelopmental challenges many survivors face. Improvements in fetal detection rates, coupled with advances in fetal ultrasound and MRI imaging, are contributing to a growing body of evidence that abnormal brain architecture is in fact present before birth in hypoplastic left heart syndrome patients, rather than being solely attributable to postnatal factors. We present an overview of the contemporary data on neurodevelopmental outcomes in hypoplastic left heart syndrome, focussing on imaging techniques that are providing greater insight into the nature of disruptions to the fetal circulation, alterations in cerebral blood flow and substrate delivery, disordered brain development, and an increased potential for neurological injury. These susceptibilities are present before any intervention, and are almost certainly substantial contributors to adverse neurodevelopmental outcomes in later childhood. The task now is to determine which subgroups of patients with hypoplastic left heart syndrome are at particular risk of poor neurodevelopmental outcomes and how that risk might be modified. This will allow for more comprehensive counselling for carers, better-informed decision making before birth, and earlier, more tailored provision of neuroprotective strategies and developmental support in the postnatal period.

Surgical outcomes for patients with CHD have traditionally been measured in terms of short-term morbidity and mortality. As survival from cardiac surgery continues to improve, robust data are emerging on the prevalence of a wide range of neurodevelopmental impairments in up to half of survivors,^1^ with univentricular conditions such as hypoplastic left heart syndrome among the highest risk.^1–6^ Indeed, the burden of impairments in these patients has prompted some groups to suggest that neurodevelopmental outcomes should be one of the primary outcome measures in clinical decision making for patients with CHD.^7^

The potential causative and contributory factors are myriad. Many CHDs require corrective or palliative surgery in the neonatal period, and the use of cardiopulmonary bypass, deep hypothermic circulatory arrest, and various surgical and postoperative factors have all been implicated in contributing to adverse neuro-developmental outcomes.^3,8–10^ The palliative approach used in univentricular conditions necessitates multiple obligatory surgeries, persistently abnormal haemo-dynamics, and lifelong cardiovascular morbidity.^11^ Chromosomal and other syndromic conditions associated with CHD are independently associated with impaired neurodevelopmental status.^12^ Other genetic factors, for example, expression of neuroprotective apolipoprotein E, may help modulate brain injury following surgery in certain patients.^13^ Socio-economic and parental factors also influence later intellectual development.^14,15^ Increasingly, however, both preoperative and prenatal brain dysgenesis, immaturity, and white matter injury are being recognised in patients with CHD.^16,17^

In the last few decades, major advances have been made in diagnosing structural heart defects before birth, and anatomical variables that are likely to contribute to poor surgical outcomes are more effectively diagnosed than ever before.^18^ The ability to identify fetuses and neonates with pre-intervention brain abnormalities, reduced cerebral blood flow, or other risk factors associated with later neurological impairments may also help identify subgroups of patients at high risk of poor neurodevelopmental outcomes. This could have important consequences for prenatal counselling, which often focusses on fetal risk factors affecting surgical mortality, even though these may in fact have little effect on parental decision making.^19^ It may also help identify infants at increased risk in the early neonatal and perioperative period, in whom novel neuroprotective techniques may be of particular benefit.^20^

This manuscript reviews existing data regarding neurodevelopmental outcomes in patients with hypoplastic left heart syndrome, with particular attention to factors encountered in the fetus or neonate before surgical intervention that may have a bearing on later brain development.

## Hypoplastic left heart syndrome and neurodevelopmental outcomes

Hypoplastic left heart syndrome is a spectrum of congenital cardiac anomalies in which the left-sided heart chambers are small or absent with a hypoplastic ascending aorta and arch. This results in the inability of the left heart to support the systemic circulation in postnatal life.

Norwood et al21 performed the first successful staged surgical palliation in a group of patients operated on from 1979 to 1981; however, in most centres, it was well over a decade before the procedure was performed in significant numbers. Thus, an increasing number of patients with hypoplastic left heart syndrome are now reaching school age and early adulthood, with consequent recognition of the significant neurodevelopmental challenges many survivors face. Over 30% of infants with hypoplastic left heart syndrome experience moderate to severe neuro-developmental impairments,^4^ including deficits in gross and fine psychomotor development, hypotonia, behavioural problems, microcephaly, and global developmental delay.^22^ School-aged children often manifest motor deficiencies such as poor balance, coordination, and dexterity; behavioural issues such as shyness or inattention; and significant cognitive challenges.^22^ Indeed, up to a third of these children will receive some form of special education, with around one in five scoring an intelligence quotient (IQ) of <70.^2^ A similar picture is emerging from the growing cohort of patients surviving into adulthood.^11^ Patients with hypoplastic left heart syndrome appear to be significantly more affected than patients with other univentricular conditions in terms of both neurocognitive^3^ and educational and behavioural difficulties.^5^

Despite recent advances in surgical and bypass techniques used in the treatment of hypoplastic left heart syndrome such as off-bypass neonatal palliation, anterograde regional cerebral perfusion during cardiac arrest, and near-infrared cerebral saturation monitoring,^9,23^ neurodevelopmental outcomes following the Norwood procedure have remained relatively static.^24,25^ As early as the 1990s, however, evidence began to emerge of clinical neurological and electroencephalographic impairments in newborn babies with CHD even before intervention,^26,27^ which were strongly linked to later neuromotor and cognitive impairments, functional limitations, and impaired quality of life.^1,27^ There is now a growing body of evidence that the neurodevelopmental outcomes for hypoplastic left heart syndrome patients are not solely determined by clinical, surgical, and interventional factors after birth; a significant burden of pre-existing brain abnormalities exists even before any attempts at surgical palliation.

## Effects of hypoplastic left heart syndrome on fetal circulation

**Figure 1:**
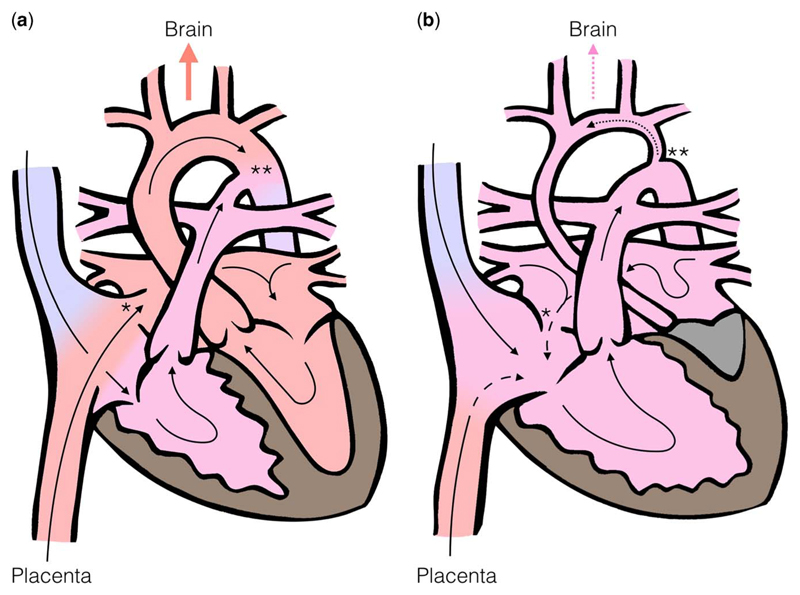
In the normal fetal circulation (a), oxygenated, blood is shunted to the left heart via the foramen ovale (*), allowing nutrient and oxygen rich blood from the placenta to be preferentially directed to the cerebral circulation. In the fetus with HLHS (b) the left sided structures are underdeveloped, leading to reversed flow at the foramen ovale (*) and aortic isthmus (**), which may be severely hypoplastic. This leads to reduced oxygen and substrate delivery to the developing brain.

In the normal fetus, both the right and the left sides of the heart support the systemic circulation, atrial and arterial shunts are present, and the placental circulation provides both oxygenation and nutritional support (Fig 1a).^18^ Placental blood is preferentially streamed via the foramen ovale to the left heart, which supplies the superior portion of the systemic circulation, delivering oxygen-rich and nutritionally rich blood to the ascending aorta, carotid arteries, and the developing brain.

The effects of abnormal cardiovascular anatomy on the fetal circulation have been considered in detail since the 1970s.^28^ Depending on the nature of the anatomical disturbance, flow rates in the ascending aorta can be increased or decreased, and the preferential shunting of oxygenated blood to the head and neck vessels may be disrupted.

In the case of hypoplastic left heart syndrome, flow through the often profoundly underdeveloped left ventricle is either reduced or absent. The ascending aorta and the aortic arch are also frequently hypoplastic and dependent on retrograde filling via the arterial duct (Fig 1b). In addition, the obligatory intracardiac mixing of oxygenated and deoxygenated blood and loss of venous streaming results in blood reaching the brain having a lower oxygen and nutritional content than in the normal fetal circulation.^29^

## Effects of hypoplastic left heart syndrome on fetal brain development

Normal fetal brain growth and development is a function of adequate oxygen and substrate delivery, and the fetus has complex mechanisms for autoregulation of cerebrovascular resistance to increase oxygen delivery and meet cerebral energy requirements.^30^

Intrauterine growth restriction31 and particularly microcephaly32 are associated with hypoplastic left heart syndrome, with the latter potentially reflecting abnormal brain development in utero. In hypoplastic left heart syndrome, the entire cerebral circulation is dependent on the retrograde flow from the arterial duct through the aortic isthmus, with potential restriction to blood flow to the aortic arch and head and neck vessels (Fig 1b).^33^ Microcephaly is known to be associated with reduced cerebral blood flow before birth,^34,35^ and has also been independently associated with a diminutive ascending aorta in hypoplastic left heart syndrome patients.^7^ Cerebral autoregulatory mechanisms offer further insight: during periods of hypoxic stress, the normal fetus compensates by reducing cerebrovascular resistance to re-distribute blood flow to the upper body and brain. This mechanism can be identified prenatally by Doppler ultrasound interrogation showing increased diastolic velocities in the cerebral arteries and reduced diastolic velocities in the descending aorta and umbilical artery.^36^ Normative indices of resistance and pulsatility have been established, and z-scores are available.^30^ Several studies have shown lower than normal cerebral artery/umbilical artery resistance ratios in patients with hypoplastic left heart syndrome,^37,38^ suggesting activation of brain-sparing mechanisms in an attempt to increase cerebral blood flow. Indeed, the presence of reversed flow across the aortic isthmus may in itself be an important independent factor in triggering this response.^39^ Maintaining the ability to re-distribute blood flow in this manner may be important; in one retrospectively analysed study of the echocardiograms of 134 hypoplastic left heart syndrome and other single-ventricle fetuses, lower fetal cerebrovascular resistance was positively associated with better neuro-developmental scores in later life.^40^

In addition to microcephaly, both structural brain abnormalities and white matter injury have been identified in patients with hypoplastic left heart syndrome. Neuropathological studies of the brains of fetuses with hypoplastic left heart syndrome have shown structural abnormalities such as agenesis of the corpus callosum and holoprosencephaly,^41^ as well as significant white matter injuries.^32^ Prenatal and postnatal MRI studies have further defined structural abnormalities, demonstrating incomplete closure of the operculum, ventriculomegaly, and cerebral atrophy. Multiple patterns of brain injury have also been described, including haemorrhagic and thromboembolic infarction, cerebral venous thromboses, periventricular leukomalacia, and grey matter injury.^1,16,42,43,44^

Although fetal brain injuries detected by MRI have been shown to be strong predictors of neuro-developmental disability in other high-risk term and preterm newborns,^45^ precisely how preoperative brain injury correlates to later impairment in hypoplastic left heart syndrome patients is not yet clear. In the largest study to date, no direct correlation was found between preoperative brain injury across a range of CHD diagnoses and neurodevelopmental outcomes at 12 months, although over a third of patients either did not survive or failed to return for testing.^46^ Further longitudinal studies are needed, both to provide a uniform means of sub-classifying preoperative brain injuries and to correlate these with later performance across all neurodevelopmental domains.

Advanced fetal MRI techniques have offered further insights into abnormal brain development in hypoplastic left heart syndrome. MRI spin-label perfusion studies have shown significantly reduced cerebral blood flow in the months before surgery.^47^ Magnetic resonance spectroscopy enables in vivo quantification of certain chemical compounds and metabolites. Using this technique, term newborns with single-ventricle hearts were shown to have increased cerebral lactate levels, which may indicate inadequate cerebral metabolism before surgery.^16^ In addition, by measuring the levels of *N*-acety-laspartate, a marker of neuronal integrity, lactate/choline ratios, and average diffusivity measured by diffusion tensor imaging, patients with CHD have been found to have features of delayed brain maturation versus controls of around 1 month, re-asserting that inefficient cerebral substrate delivery is likely to begin before birth.^30^ Indeed, by using similar techniques on the fetal brain, prenatal MRI studies have identified that brain metabolism, growth, and maturation are particularly delayed in the third trimester, a period of increased synapse formation, myelination, and high metabolic demand. A reduction in combined ventricular output across the aortic valve was independently associated with a reduction in total brain volume, and absence of anterograde arch flow was associated with delayed brain maturity.^48^ In contrast to overt brain injury, markers of brain immaturity appear to correlate more convincingly to later neurodevelopmental status, with severity at birth shown to be a good predictor of neurodevelopmental impairment at 2 years of age after cardiac surgery.^49^ Furthermore, studies in adolescents have also shown a direct correlation between brain volume and neurodevelopmental outcomes.^50^

Finally, although it has been demonstrated that patients with delayed brain maturation are at particular risk of sustaining new white matter injuries postoperatively,^51^ pre-existing brain injury does not appear to worsen following surgery, adding more weight to the argument that surgical factors may play a lesser role than that previously suspected.^43,52^

## Opportunities for the future

Alongside exciting developments in three and four-dimensional fetal ultrasound such as spatio-temporal image correlation imaging,^53^ fetal MRI is quickly gaining ground as an important complementary imaging tool, with the additional resources required increasingly offset by a growing number of fetal indications^54^ and rapidly developing MRI infrastructure.^55^ By combining established techniques with advanced motion-correction algorithms,^56^ retrospectively gated flow measurements,^57^ and non-invasive estimates of intravascular oxygen saturations,^58^ there is the potential to generate a detailed anatomical and physiological profile of the fetal circulation in CHD.

In addition, centres performing prenatal brain imaging are enhancing our understanding of both gross brain injury and subtle patterns of disordered brain development and oxygen uptake and metabolism, which may accompany these disruptions.^58–60^ In correlating these data to later neurodevelopmental impairments, it may be possible to establish hypoplastic left heart syndrome cardiac phenotypes, patterns of disordered brain development, and categories of brain injury that confer a higher risk for later neurodevelopmental problems. Addressing modifiable risk factors via tailored neuroprotective strategies,^9^ novel monitoring techniques,^23^ and judicious attention to anaesthetic load,^46^ in conjunction with subsequent early access to developmental support programmes, could help improve longer-term outcomes.^20^

Further into the future, as we begin to understand the complex fetoplacental interactions in fetuses with CHD, manipulation of the fetal circulation via experimental interventions such as maternal hyperoxygenation may provide a non-invasive means of improving cerebral oxygen and substrate delivery in selected patients.^61^ Finally, the use of direct fetal cardiac intervention, currently being explored by a small number of centres for a limited set of indications,^62^ may develop a role as our understanding and expertise develop in this area.

## Impact on fetal counselling

Counselling the prospective parents of a fetus with hypoplastic left heart syndrome poses many challenges for physicians, counsellors, and the parents themselves. Potential management options may include a variety of approaches, including variants of the Norwood procedure, the hybrid procedure, compassionate care after birth, primary transplantation, or termination of the pregnancy. As such, an antenatal diagnosis of hypoplastic left heart syndrome demands tailored, sensitive, and intuitive counselling, relevant to each parent’s level of understanding and cultural, moral, and emotional needs.^63^ Determining fetal phenotypes at high risk not only of surgical mortality but also of poor neurodevelopmental outcome may allow for more comprehensive counselling for carers, in whom predictions based on mortality risk alone have been shown to have little effect on antenatal decision making.^19^ A more comprehensive risk profile that includes neurodevelopmental risk factors may be far more relevant for prospective parents, in terms of both antenatal decision making and longer-term expectations.

## Summary

Neonates with hypoplastic left heart syndrome have a unique set of neurological vulnerabilities before they even encounter the operating theatre, with the potential for impaired cerebral blood flow, oxygen and nutrient delivery, leading to delayed or disordered brain development, and pre-existing brain injuries. These factors are present before any intervention, and are almost certainly a substantial contributor to the many factors that may contribute to adverse neurodevelopmental outcomes in later childhood and adulthood.

The use of novel cardiac neuroimaging before and after birth is enhancing our ability to define specific hypoplastic left heart syndrome phenotypes at risk of developing significant neurodevelopmental impairments, potentially providing opportunities to tailor neuroprotective strategies, enable early access to structured neurodevelopmental support programmes, and enhance fetal counselling.
